# Supplementary material for: Rapid activation of distinct members of multigene families in Plasmodium spp
Source: Commun Biol. 2020 Jul 3;3:351. doi: 10.1038/s42003-020-1081-3 (PMC7334209; doi:10.1038/s42003-020-1081-3)
Supplement: Supplementary file 1 — Supplementary Information [file 42003_2020_1081_MOESM1_ESM.pdf]

Supplementary Figures

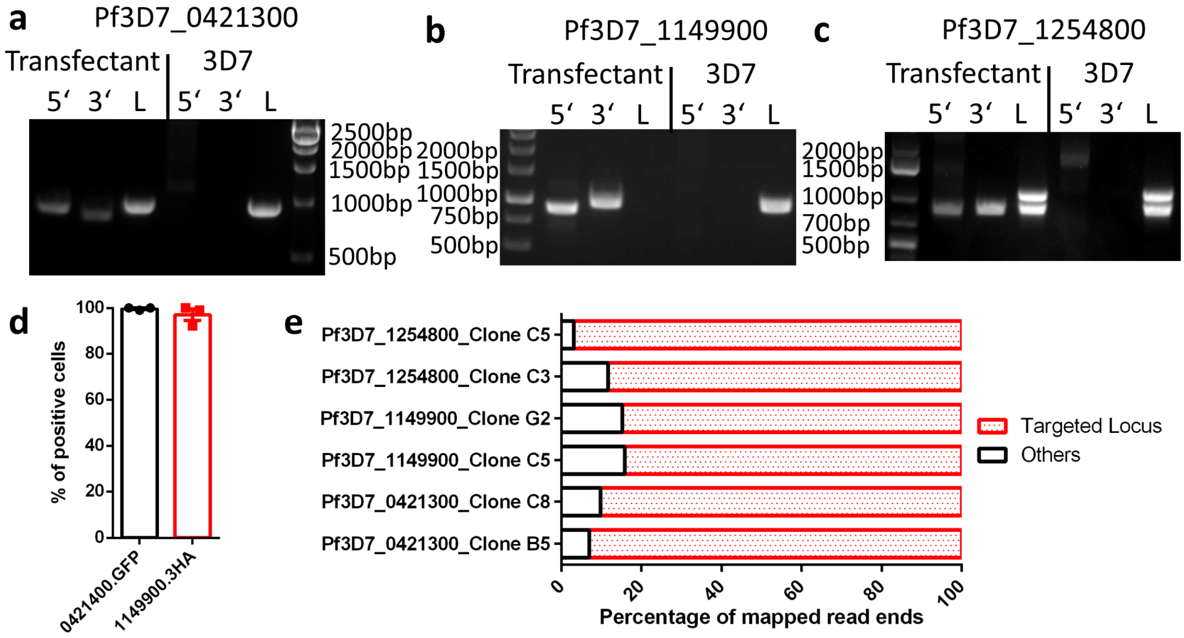

Supplementary Figure 1: Validation of correct plasmid integration into the *P. falciparum* genome. (a-c) Diagnostic PCR to verify pSLI-based integration. Primer pairs were designed according to [62] and should amplify fragments of 800-900 bps length either 5' or 3' of integration site after integration into the genome. Third primer pair (L) spans the entire locus and should disappear after integration. The primers used were the gene specific forward + plasmid reverse for the 5' arm, gene specific reverse + plasmid forward for the 3' arm and the two gene specific ones for the genomic locus. Persistance of the wild type locus after integration is due to amplification of the unmodified locus. For further validation we sent all 5' fragments for sequencing. (d) Parasitized 1149900.3HA (*stevor*) and 0421300.GFP (*var*) in immunofluorescent assays. For each cell line, 100+ parasites (at least 18hpi) were counted by three different members of the lab. Both parasite lines showed almost 100% staining for the tagged STEVOR and PfEMP1 (97 and 99.7% respectively), supporting integration of the transgene in these parasite populations. All data are presented as mean ± standard error of the mean (SEM) (e) Mapping of Nanopore read ends to the modified locus. Whole genome sequencing was performed on two clones of all thee *P. falciparum* knock in cell lines. The 200bps at each end of reads that span the plasmid were mapped to the reference genome. Percentage of reads that map to the correct locus was calculated. Reads that include the plasmid but map to any other locus within the genome are given as "others". The majority of read ends map to the targeted locus, showing correct integration of the plasmid to the locus of interest. Every read spanning the plasmid and extending beyond its boundaries was manually confirmed to be integrated into the correct, targeted locus.

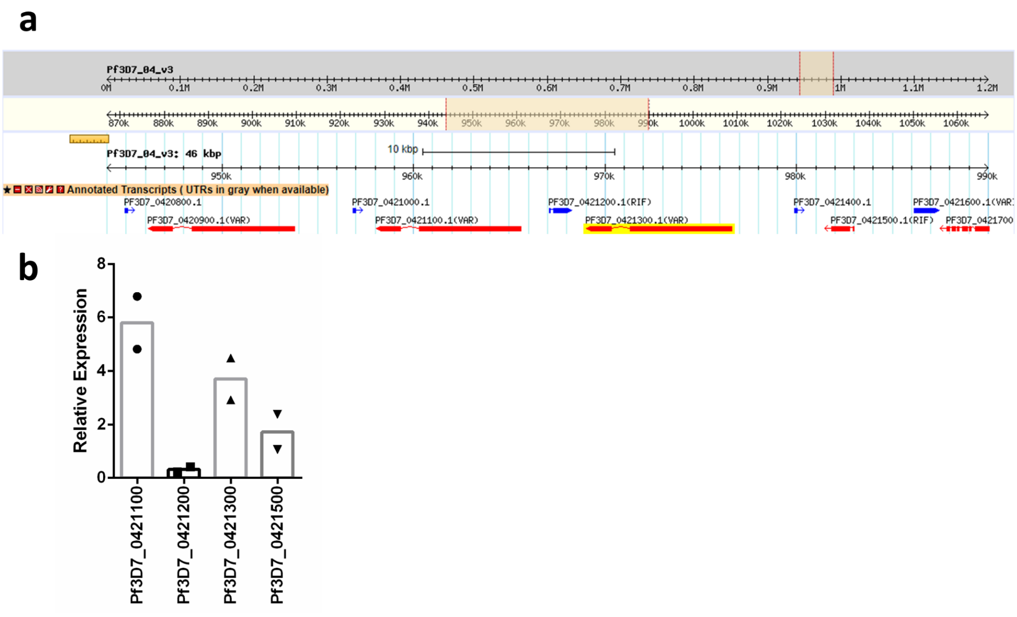

Supplementary Figure 2: Further explanations for the “open chromatin” theory. (a) The genomic locus surrounding the targeted *var* Pf3D7_0421300. Both the *var* Pf3D7_0421100 and the *rif* Pf3D7_0421500, which are oriented in a head to tail manner to the targeted *var*, show upregulation according to the microarray, while the *rif* Pf3D7_0421200, which is oriented head to head, remained silent. (b) Quantitative RT PCR validation of the 4 multigene family members differentially regulated within this genomic locus. The three genes upregulated according to the microarray show high expression levels while the non-regulated *rif* is barely detectable. All data are presented as mean. N=2

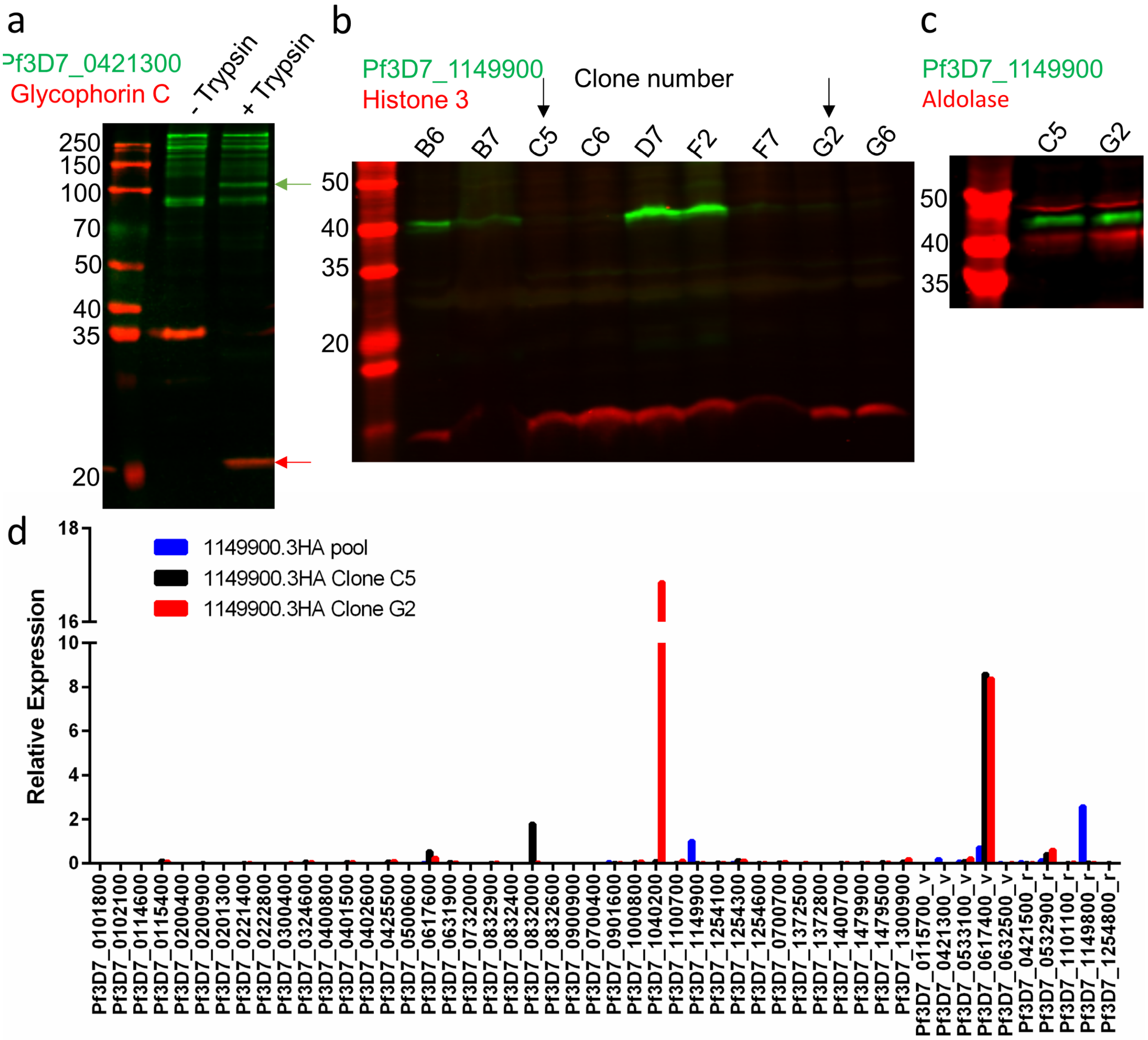

Supplementary Figure 3: Further characterization of knock-in cell lines. (a) Pf3D7_0421300.GFP is partially presented on the surface of the infected red blood cell. Percoll enriched late stage parasites expressing Pf3D7_0421300.GFP were subjected to live trypsin treatment. After treatment, an additional band corresponding to the size of PfEMP1 ATS and GFP is detectable on a western blot (green arrow). (b-d) Knock-in parasites stop expression of the targeted protein without drug pressure. Neomycin was removed from the culture medium of 1149900.3HA parasites and single clones were obtained by limiting dilution. (b) The clones obtained from limiting dilution were tested for expression of the fusion protein by western blot using Histone 3 as loading control. Four of the clones (C5, C6, G2, G6) showed no expression of the HA-tagged STEVOR. Two of the clones (C5, G2, highlighted with black arrows) were chosen for further studies. (c) Expression of the fusion protein can be recovered upon addition of the drug. Neomycin was added back to the culture medium of the non-expressing clones C5 and G2. After an initial drop in parasitaemia, late stage parasites were collected and analysed using western blot. Expression of the fusion protein could be detected in both clones. (d) Clonal dilution identified parasites expressing different *stevor*. The two chosen clones were tested for their expression of all 40 annotated *stevor* genes and the previously tested *var* and *rif* (labelled with v or r respectively). In accordance to the western blot results, neither of the clones shows detectable levels

of Pf3D7_1149900 expression. Furthermore, expression of the *rif* Pf3D7_1149800 dropped below detection limits as well, in line with the hypothesized co-expression of these two genes. Expression of the *var* Pf3D7_0617400 further increased compared to the unselected pool. Expression of only the targeted gene can be detected in the pool (black arrow). Clone C5 expresses two *stevor* (Pf3D7_0832000 and Pf3D7_0617600) while clone G2 expresses only Pf3D7_1040200 at very high levels. Considering that *stevor* expression *in vitro* is generally reduced, the high expression levels observed in clone G2 might more closely reflect *in vivo stevor* expression. N=1

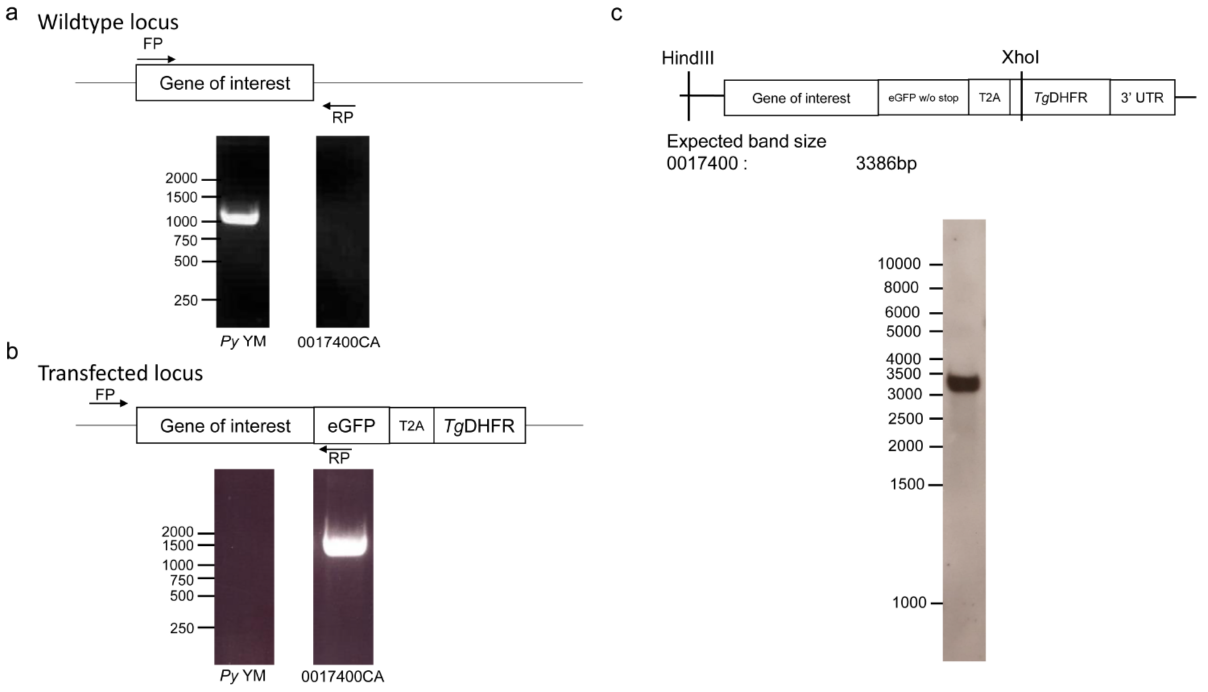

Supplementary Figure 4: Validation of integration of PYYM_0017400. a) Diagnostic PCR showing the loss of the wildtype locus after integration of the transgene. b) Diagnostic PCR showing the integration of the skip peptide construct into the genome of *P. yoelii*. FP indicated forward primer binding region and RP indicated reverse primer binding region. c) Southern blot analysis of genomic DNA digested with HindIII and XhoI and hybridized with probe specific to the GFP sequence.

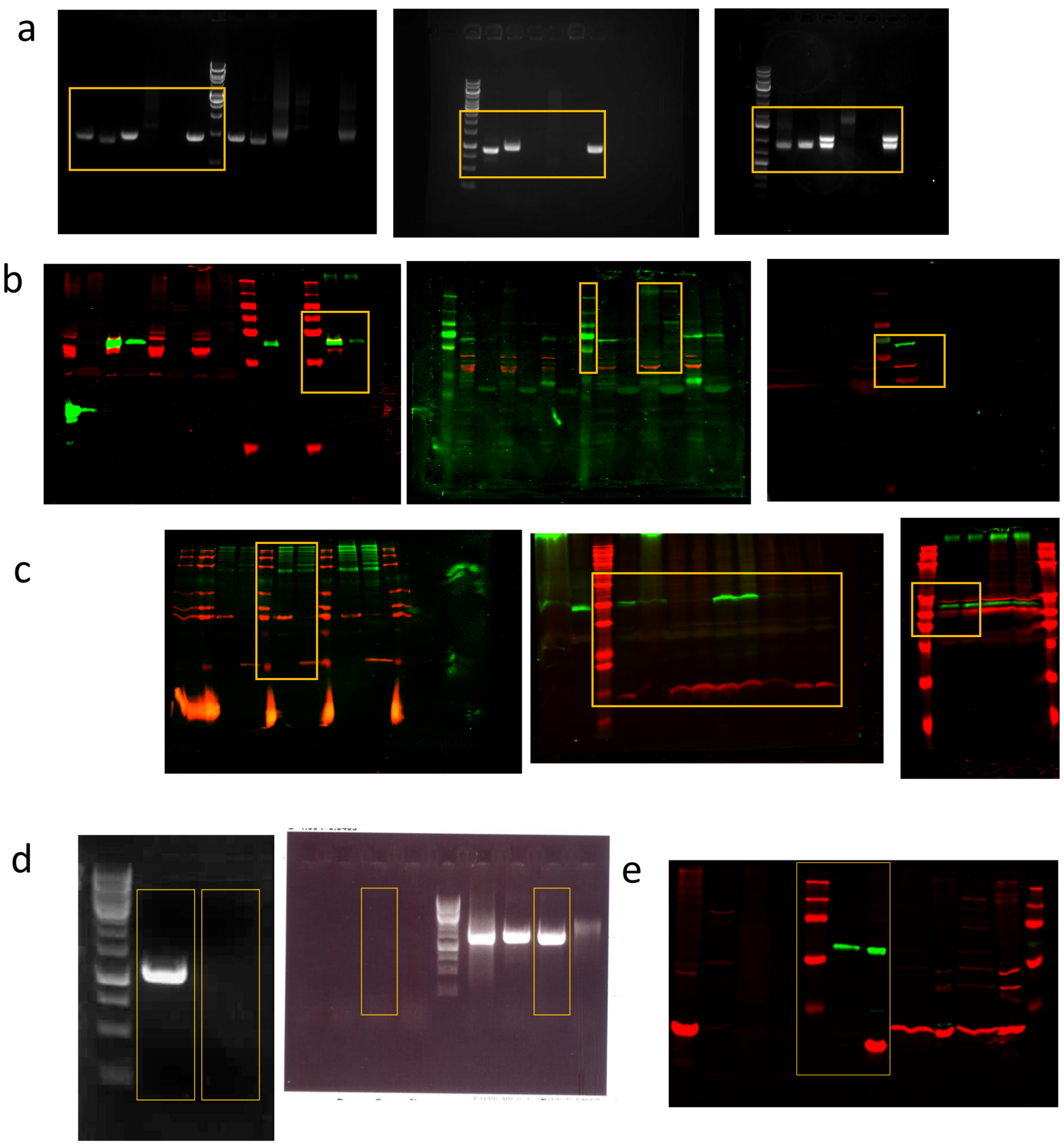

Supplementary Figure 5. Full Western blot images of Figure 2 (b), Figure 5 (e) and Supplementary Figure 3 (c). Full gel images of Supplementary Figure 1 (a) and 4 (d)

Supplementary Tables

	Average transcript per million counts of *yir* genes			
	PY17X_1100077	PY17X_0401000	PY17X_0114700	PY17X_1100081
Parental YM	5.95	3.46	1.05	1.04
SC1	513.34	210.90	221.52	38.16
SC3	557.94	547.61	418.62	38.52

Supplementary Table 1: Average transcript per million counts of upregulated *yir* genes in RNAseq.

Gene ID	Fwd primer	Rev Primer
Pf3D7_0421300 (var)	GTATGTGTACATTTATGGCATAATGTAGTCGGG	CCGACAAATATAACCACTATACAAAAAAAAACACATG
Pf3D7_1149900 (stevor)	CTCATTCAAAACAAGACCAAAAGAGCGA	CGTGCAATCTTATGTTTTGAAATAATTATTGTC
Pf3D7_1254800 (rifin)	GTGCGAATTATATATGTCCAATTATGACAACGATCC	GAAAAACAGTACATGTACCAAACATCCTACC
3xhemagglutinin tag	GGGCGTACGTACCCATACGACGTC	GGGGTCGACGGCATAATCTGGAAC
T2A/DHFR	ATAGGGCCCGGCGCCGTCGACGGAGAAGGAAGAGG	AGTGTATGCGGCCGCGGATCCAACGGGATCAAAGCT
Intermediate insert	ACCAAGCTTGATATCCCTAGGAAAGTTACAGACCC	TATGGGCCCTTTGATGGTATATCCAAAAAAAAAGG
eGFP	ATAGGGCCCGTGAGCAAGGGCGAGGAGCTG	TATGGCGCCCTTGTACAGCTCGTCCATG

Supplementary Table 2: List of cloning primers used

Gene ID	Fwd primer	Rev Primer
Pf3D7_0421300 (var)	CTCGCGGCCGCTAACACAACCGAATACTTTATATTTTAAT	AGCACGCGTTATATCCCACACATCTCCTATAGGATAT
Pf3D7_1149900 (stevor)	CTCGCGGCCGCTAAGCACAAACCCAAAACCATAATCCGC	AGCACGCGTCTTACATAAATGTTTCTTGCATTCATGTTTCCATG
Pf3D7_1254800 (rifin)	CTCGCGGCCGCTAATGATGCAACAATTTCATG	AGCACGCGTTTCTTTTAATAATTTTATATATTGGAG
Universal plasmid primer	TAT TTA GGT GAC ACT ATA GAA TAC TCG	CTA CCT GCA CCT CCA GCA CCA GC
PY17X_1100077 (yir- integrated locus)	ATGAGGAAACATTTACCCGATAAATTAGG	CCGGTGAACAGCTCCTCGCCCTTGCTCAC
PY17X_1100077 (yir- wildtype locus)	ATGAGGAAACATTTACCCGATAAATTAGG	ACGAGATGGTACATATATTC
Southern blot probe	ATAGGGCCCGTGAGCAAGGGCGAGGAGCTG	TATGGCGCCCTTGTACAGCTCGTCCATG

Supplementary Table 3: List of integration validation primers used

Gene ID	Fwd primer	Rev primer
Pf3D7_0115700 (var)	AATATTGCGATCTTAATAGGTATGATTGCG	GCACCACCTGTTATTTCGCTTTTG
Pf3D7_0421300 (var)	GCTAACCAATATAAAGATGACTTGAAAGG	AAAACGCTCTACATCGTTTCCG
Pf3D7_0533100 (var)	TGGCGAAGGTATAATGATAAAGG	TACTGTTACTACATTCAGATATTTGTCC
Pf3D7_0617400 (var)	CATACAGTAGTGCCAAGGACC	GCGCAAGGATTATTTTTATCAAAACC
Pf3D7_0632500 (var)	CGCTGGATGAAAGTGTCAAAG	TAACAAGGATTCTCTTTACCACC
Pf3D7_0421100 (var)	GGAAAATATCAACTAACTAATCATGG	GCATTCCTTAATTTATAATAATTTGGATC
Pf3D7_0421500 (rifin)	CACCAACTACTAAGTCACGATT	ATGCCTCTAACTTTTCTGTTAATTCC
Pf3D7_0532900 (rifin)	CGAAATGAACCATCACAACACA	CTGTTTTTGTTCACACGTTCATTG
Pf3D7_1101100 (rifin)	AATTATACGCACCATCTAACTATGAC	CATTTTGTATATCAGTTTGTAACGTTAAGA
Pf3D7_1149800 (rifin)	CCATACATCTCACCACATACAC	CAATAATTTTTTTTATATCTTTGTCACATTGTTC
Pf3D7_1254800 (rifin)	CGCGACATTGAACACTAATATAACTAATGAG	TCAATTGTTTTCGCAATAAATGCGTTG

Pf3D7_0421200 (rifin)	ATGTCACAAAGATTGCTCG	GCAATGCTGTTTTTTATAGCTT
Pf3D7_0101800 (stevor)	TGATGCCCTTGCTAGTTATGC	CAGAAGTTGCAGCAGGTACTGT
PF3D7_0102100 (stevor)	GGTTTGGCAAAGGCAAAATA	CAAGCTGAACTACCAGCTTCAA
PF3D7_0114600 (stevor)	CAGCTATTCAAGCAGGTGCTAA	CAATACCACAACCTCCAGGAA
Pf3D7_0115400 (stevor)	GGCTGCCTTTGATACCTTGA	TGTACCGCCTGCAAACATAG
PF3D7_0200400 (stevor)	GCCCTAAGTGCTGTTGCTTC	CACCAACAACACATGCTTTCA
PF3D7_0200900 (stevor)	CTGCTGCCATTGCTACCTTT	AGCAGCACCAGATGCACATA
PF3D7_0201300 (stevor)	GCAAAATCTGCTGCCCTTAC	ACCATTTGCACAGGTTCCAC
PF3D7_0221400 (stevor)	TAAATTTACGAAGGCGCTTG	CCAGAGCTGCAATACCATAA
PF3D7_0222800 (stevor)	TGCTGCTGTCACTTCTAGCTTT	CTACACCTCCTGCTGCAGTAAC
PF3D7_0300400 (stevor)	AAAGGTGCTGCTATTTCTACCG	CAGCAGCACAACCTACTGTACC
PF3D7_0324600 (stevor)	TGCATGCTGCTAAAGTTGCT	TTGGTTGCACAAAGAACTGACT
PF3D7_0400800 (stevor)	TGGCAGCTACCAAAGCTACA	TCAGGACCCCAAGCTGTAATA
PF3D7_0401500 (stevor)	AAATGCGCATCCTCTATCACT	AGTTCCGACCCCATCACTAA
PF3D7_0402600 (stevor)	TTTATGGTATTAGCGCTGCAAG	GCGCTTTCTATTGATGTTTGAG
PF3D7_0425500 (stevor)	TCATCATCCCAATTGACGAA	CTTCATATTCGACGGACGTT
PF3D7_0425500 (stevor)	AATGCGTTGCCCTCTATATC	TCATATTCGACGGACGTTAT
PF3D7_0500600 (stevor)	GCAAAGGTTCTTGCTGGTGT	GCTATTCTCATAGCATCCCAATG
PF3D7_0617600 (stevor)	CTGCACCTTATGATGCTTGTGT	AAACAGTTCCGGCCATATCA
Pf3D7_0631900 (stevor)	GTTGATGCCATCCTTCCTGT	ACGCTTCGGTTGCTTTAAGA
Pf3D7_0700400 (stevor)	GCAAAAGCTGCTATCCTTGG	ACACCACCTGATGCACTAACTG
PF3D7_0700700 (stevor)	CCTGTTGGTTTATGGTCTCC	ATGTTTTCGTCTGTTGCGTA
PF3D7_0732000 (stevor)	TTGCAGAAGTGCCTAAGAATTG	GCACTAACAGTAGTTGCTTCTCCT
PF3D7_0832000 (stevor)	ACAGAATTCGCTGGTAATGT	AACTTGCAGCAAAGAAACTC
PF3D7_0832400 (stevor)	AAGTGAGATGTTCCCGTGGT	TGGACAGTAAAGTGCGCAAG
PF3D7_0832600 (stevor)	AATTGTCCCAAACCCTTGTC	AGCCTTCGTAGCCATTGAAC
PF3D7_0832900 (stevor)	TACGTGCGCATCCCATATTA	TTCCGACTAAATCACCAGCA
PF3D7_0900900 (stevor)	TTGCAGAAGTGCCTAAGAATTG	CATAGCAGCACTTGCAGCAG
PF3D7_0901600 (stevor)	GGCTGCACTTGCTTACTTTTC	TAGCACATGTTGCACCTCCT
PF3D7_1000800 (stevor)	TGGGGCTCCTACATTAAGTCA	CACGACAATTGCCATACCAT
PF3D7_1040200 (stevor)	AGGTGCTCTTGCTGAGTATGC	CAAATTTCAGTGCCTGCTTG
PF3D7_1100700 (stevor)	AGAACGTTGTGTTGGAGGTGT	GGAAGCCATAAACCAACAGG
Pf3D7_1149900 (stevor)	TGACGTTGCTGCCTTGAAATA	ACCAACGCCTGCTTGAATAG
PF3D7_1254100 (stevor)	TGCGTGCAAATCCTCTATCA	AAACAAGCTGTACTACCTGC
PF3D7_1254300 (stevor)	GCTAAAACGGCTGCCCTAA	CACCACTTGCACAAAATCCA
PF3D7_1254600 (stevor)	TCGGGCATGAGTCCCAATTT	TTAGGGCAGCCGTTTTAGCA
PF3D7_1300900 (stevor)	GCCGTTGCTTCTCTTGTTTATT	ATCAGTAGCACCGGCAAGAG
PF3D7_1372500 (stevor)	CCTTACTAAATTGACCCAAGC	CTTTTTCTTCTTTTACGCAGC
PF3D7_1372800 (stevor)	CTGCTAAAACGTGTGCGTCT	ATTTGCAGCAGGCAAAACTA
PF3D7_1400700 (stevor)	CTTCCGTTGGACCACCTTAT	CTTGCAGCAGTTCCGGTTAT
Pf3D7_1479500 (stevor)	GCAAAAGTTGCTGTCATTGG	GAGCCTGCTGCATTAACTGA
PF3D7_1479900 (stevor)	CATGAAGGCTGTTGCTGATTAT	ATCAGTAGCACCCTCAACACAT
PF3D7_0717700 (serine tRNA ligase	AAGTAGCAGGTCATCGTGGTT	TTCGGCACATTCTTCCATAA
PY17X_0401000 (yir)	TGATAGTAGAGATCCTAGTAAGTC	ATTTGGCTTGTTTGCATCAAGTTC
PY17X_0102100 (yir)	ACAAACTTAATGAAAATGTACCCG	ATGCTTTCCATATAATTTATCACC
PY17X_1100077 (yir)	GATACTGATTTCGATAAAAATACGGC	ATCATTTATTTTAGTGAATTTGTTCCC
PY17X_0114700 (yir)	GGTTTTTCAGCTGGTCAAGATG	ATTTTATGGTTAGAGTACTCCACG
PY17X_1400400 (yir)	TAATATCCTCCAGAAAGTAACGAAG	CATCAAACAAAAATAAACATGCAGC
Py17X_1046201 (yir)	TGAGGAAGCATTTCCCTGATG	ATCCAGTATTGCCTTTAATTGGG
Py17X_1046301 (yir)	ATGTATGGGATGATTTCCCCG	AACCAGAAGGCCCAAAAAATTG
PY17X_0712100 (Heat Shock Protein 70)	GCTGATAACCAACCAGGAGTC	CAGGTGGGATACCATCTAAATG

Supplementary Table 4: List of RT-PCR primers used
