# Supplementary material for: Rapid activation of distinct members of multigene families in Plasmodium spp
Source: Commun Biol. 2020 Jul 3;3:351. doi: 10.1038/s42003-020-1081-3 (PMC7334209; doi:10.1038/s42003-020-1081-3)
Supplement: Supplementary file 2 — Description of Additional Supplementary Files [file 42003_2020_1081_MOESM2_ESM.pdf]

**Description of Additional Supplementary Files**

File Name: Supplementary Data 1
Description: All source data underlying the graphs presented in the main Figures 3d, 4 and 6b as well as Supplementary Figure 1d and e, 2b and 3d. Each sheet is labelled with the figure the raw data corresponds to.
