# Supplementary material for: Rapid activation of distinct members of multigene families in Plasmodium spp
Source: Commun Biol. 2020 Jul 3;3:351. doi: 10.1038/s42003-020-1081-3 (PMC7334209; doi:10.1038/s42003-020-1081-3)
Supplement: Supplementary file 5 — Reporting Summary [file 42003_2020_1081_MOESM5_ESM.pdf]

nature research

Corresponding author(s): Peter R. Preiser

Last updated by author(s): Jun 9, 2020

Reporting Summary

Nature Research wishes to improve the reproducibility of the work that we publish. This form provides structure for consistency and transparency in reporting. For further information on Nature Research policies, see our Editorial Policies and the Editorial Policy Checklist.

Statistics

For all statistical analyses, confirm that the following items are present in the figure legend, table legend, main text, or Methods section.

n/a	Confirmed	
☐	☒	The exact sample size (*n*) for each experimental group/condition, given as a discrete number and unit of measurement
☐	☒	A statement on whether measurements were taken from distinct samples or whether the same sample was measured repeatedly
☐	☒	The statistical test(s) used AND whether they are one- or two-sided<br>*Only common tests should be described solely by name; describe more complex techniques in the Methods section.*
☒	☐	A description of all covariates tested
☒	☐	A description of any assumptions or corrections, such as tests of normality and adjustment for multiple comparisons
☐	☒	A full description of the statistical parameters including central tendency (e.g. means) or other basic estimates (e.g. regression coefficient) AND variation (e.g. standard deviation) or associated estimates of uncertainty (e.g. confidence intervals)
☐	☒	For null hypothesis testing, the test statistic (e.g. *F*, *t*, *r*) with confidence intervals, effect sizes, degrees of freedom and *P* value noted<br>*Give P values as exact values whenever suitable.*
☒	☐	For Bayesian analysis, information on the choice of priors and Markov chain Monte Carlo settings
☒	☐	For hierarchical and complex designs, identification of the appropriate level for tests and full reporting of outcomes
☒	☐	Estimates of effect sizes (e.g. Cohen's *d*, Pearson's *r*), indicating how they were calculated

Our web collection on statistics for biologists contains articles on many of the points above.

Software and code

Policy information about availability of computer code

Data collection	Microarray: Tecan PowerScanner V1.2<br>qPCR: Applied Biosystems 7500 Software V2.3<br>Andor Zyla sCMOS<br>LiCor Odyssey CLx<br>Illumina MiSeq<br>Oxford Nanopore GridION
Data analysis	GenePix Pro 3 (Axon Instruments, Union City, California, United States)<br>Kallisto windows v0.44<br>deseq2<br>R version 3.4<br>usegalaxy.eu workpackage<br>GraphPad Prism V6<br>Microsoft Excel<br>ImageJ 1.52h

For manuscripts utilizing custom algorithms or software that are central to the research but not yet described in published literature, software must be made available to editors and reviewers. We strongly encourage code deposition in a community repository (e.g. GitHub). See the Nature Research guidelines for submitting code & software for further information.

Data

Policy information about availability of data

All manuscripts must include a data availability statement. This statement should provide the following information, where applicable:

- Accession codes, unique identifiers, or web links for publicly available datasets
- A list of figures that have associated raw data
- A description of any restrictions on data availability

All relevant data are within the paper and its Supporting Information files. The microarray data discussed in this publication have been deposited in NCBI's Gene Expression Omnibus [89] and are accessible through GEO Series accession number GSE128123 (https://www.ncbi.nlm.nih.gov/geo/query/acc.cgi?acc=GSExxx). The whole genome sequencing data have been deposited with links to BioProject accession number PRJNA637985 in the NCBI BioProject database (http://www.ncbi.nlm.nih.gov/bioproject/637985).

Field-specific reporting

Please select the one below that is the best fit for your research. If you are not sure, read the appropriate sections before making your selection.

☒ Life sciences ☐ Behavioural & social sciences ☐ Ecological, evolutionary & environmental sciences

For a reference copy of the document with all sections, see nature.com/documents/nr-reporting-summary-flat.pdf

Life sciences study design

All studies must disclose on these points even when the disclosure is negative.

Sample size	Microarray: Samples were taken from the same flask 18, 24 or 30 hours post invasion.<br>qRT-PCR: Parasites were grown in the blood of 3 different donors in parallel for 3 cycles (Figure 3d, Supplementary Figure 2b). Single samples were taken for Supplementary Figure 3d. Single Cultures were treated and harvested in parallel.<br>For P.yoelii, 3 mice were infected per transfectant and harvested in parallel.<br>RNAseq: 3 mice per transfectant for the RNAseq. RNAseq samples were prepared from 2 clones of the same construct.
Data exclusions	Microarray: Only multigene members with less than 2 datapoints missing among the 12 samples were included in the analysis.<br>RNAseq: Only estimated counts of more than 20 were selected for further analysis. We want to see the significant difference between the transfectants and the wildtype therefore we consider those transcripts with less than 20 counts as background.<br>qRT-PCR: One replicate used for Supplementary Figure 2b has been excluded as one of the genes showed several fold higher expression (Pf3D7_0421300), resulting in excessively large error bars.
Replication	Microarray: Only one replicate for each of the samples. qPCR served as a validation of this dataset<br>qPCR : 3 replicates for each P.falciparum, P. yoelii. cell line<br>RNAseq: 3 biological replicates were carried out for each transfectant.
Randomization	The study is not random. The study was specifically designed for the analysis of the differences in the transcription level of the transfectant and the wildtype parasites.
Blinding	Blinding is not relevant to current study because we need to address whether there is any transcriptomic difference between the wildtype and the transfectants.

Reporting for specific materials, systems and methods

We require information from authors about some types of materials, experimental systems and methods used in many studies. Here, indicate whether each material, system or method listed is relevant to your study. If you are not sure if a list item applies to your research, read the appropriate section before selecting a response.

Materials & experimental systems

n/a	Involved in the study	
☐	☒	Antibodies
☐	☒	Eukaryotic cell lines
☒	☐	Palaeontology and archaeology
☐	☒	Animals and other organisms
☒	☐	Human research participants
☒	☐	Clinical data
☒	☐	Dual use research of concern

Methods

n/a	Involved in the study	
☒	☐	ChIP-seq
☒	☐	Flow cytometry
☒	☐	MRI-based neuroimaging

Antibodies

Antibodies used	Mouse anti GFP antibodies mouse IgG1κ (clones 7.1 and 13.1) cat# 11814460001<br>Rabbit anti aldolase (generated by Agrisera)<br>Rabbit anti Exp2<br>Rabbit anti GAP45<br>Rat anti HA rat IgG1 (clone 3F10), Sigma Aldrich, cat# 11867423001<br>Rabbit anti-sEMP1<br>Alexa 594 goat anti-rabbit, Thermofisher, A11037, lot982447<br>Alexa Fluor® 594-AffiniPure Goat Anti-Rat IgG (H+L), Jackson immune research lab, cat# 112-585-062<br>IRDye680rd donkey anti-rabbit, licor, 925-68073, lot: #c60831-06<br>IRDye800cw goat anti-mouse, licor, 925-32210, lot #C60726-03<br>IRDye800cw anti-rat, licor, licor, 926-32219<br>Alexa 488 goat anti-rabbit, life tech, A-11008<br>Alexa 488 goat anti-mouse, life tech IgG A11029, lot1705900
Validation	Mouse anti GFP:<br>Anti-GFP is tested for functionality and purity relative to a reference standard to confirm the quality of each new reagent preparation. Both Anti-GFP mouse monoclonal antibodies (Clones 7.1 and 13.1) are >95% pure as determined by SDS-PAGE and ion-exchange HPLC analyses.<br>Rabbit anti aldolase:<br>Used and validated in Yam et al 2016<br>Rabbit anti Exp2:<br>Used and validated in Siau et al 2016<br>Rabbit anti GAP45<br>Used and validated in Baum et al 2006<br>Rat anti HA:<br>Anti-HA, High Affinity recognizes the 9-amino acid sequence YPYDVPDYA, derived from the human influenza hemagglutinin (HA) protein. This epitope is also recognized in fusion proteins regardless of its position (N-terminal, C-terminal or internal).<br>Rabbit anti HA:<br>Anti-HA is produced in rabbit using a synthetic peptide corresponding to amino acid residues of the human Influenza hemagglutinin (HA), conjugated to KLH. The antibody is affinity-purified on the immobilized immunizing peptide.<br>Anti-HA antibody is specific for N- or C-terminal HA-tagged fusion proteins. The product may detect cross-reacting bands in certain mammalian cells.

Eukaryotic cell lines

Policy information about cell lines

Cell line source(s)	MR4 - BEI Resources
Authentication	None of the cell lines were authenticated
Mycoplasma contamination	P. falciparum in vitro culture was tested and showed very low levels of mycoplasma
Commonly misidentified lines<br>(See ICLAC register)	*Name any commonly misidentified cell lines used in the study and provide a rationale for their use.*

Animals and other organisms

Policy information about studies involving animals; ARRIVE guidelines recommended for reporting animal research

Laboratory animals	6 - 8 weeks male Balb/c mice
Wild animals	Did not involve wild animals.
Field-collected samples	Study did not include samples from the field
Ethics oversight	This study was carried out in strict accordance with the recommendations of the NACLAR (National Advisory Committee for Laboratory Animal Research) guidelines under the Animal & Birds (Care and Use of Animals for Scientific Purposes) Rules of Singapore.<br>The protocol was approved by the by the Institutional Animal Care and Use Committee (IACUC) of the Nanyang Technological University of Singapore (Approval number: ARF SBS/NIE-A-0379).

Note that full information on the approval of the study protocol must also be provided in the manuscript.
